# Effects of Varied Cleaning Methods on Ni-5% W Substrate for Dip-Coating of Water-based Buffer Layers: An X-ray Photoelectron Spectroscopy Study

**DOI:** 10.3390/nano2030251

**Published:** 2012-08-09

**Authors:** Vyshnavi Narayanan, Els Bruneel, Ruben Hühne, Isabel van Driessche

**Affiliations:** 1 Sol-Gel Centre for Research on Inorganic Powders and Thin Film Synthesis SCRiPTS, Department of Inorganic and Physical Chemistry, Ghent University, Krijgslaan 281-S3, Gent B-9000, Belgium; Email: els.bruneel@ugent.be (E.B.); isabel.vandriessche@ugent.be (I.V.D.); 2 IFW Dresden, Helmholtzstrasse 20, Dresden 01069, Germany; Email: r.huehne@ifw-dresden.de

**Keywords:** X-ray photoelectron spectroscopy, cleaning of Ni-5% W substrate, depth profile studies, thin films and coatings

## Abstract

This work describes various combinations of cleaning methods involved in the preparation of Ni-5% W substrates for the deposition of buffer layers using water-based solvents. The substrate has been studied for its surface properties using X-ray photoelectron spectroscopy (XPS). The contaminants in the substrates have been quantified and the appropriate cleaning method was chosen in terms of contaminants level and showing good surface crystallinity to further consider them for depositing chemical solution-based buffer layers for Y_1_Ba_2_Cu_3_O_y_ (YBCO) coated conductors.

## 1. Introduction

Second generation superconductors employing RABiTS (Rolling-Assisted-Biaxially-Textured-Substrates) offer a viable way to develop superconductor wires for commercial use. Coated conductors are based on the deposition of (0 0 l) oriented buffer layers and the c-axis oriented YBa_2_Cu_3_O_7−x_ (YBCO) films on these nickel alloy substrates. Researchers have noticed that the surface properties of the nickel surface play a crucial role in making it conducive to epitaxial film deposition [1]. The crucial factor to obtain a superconducting YBCO thin film is dependent on the ability to deposit smooth, dense, crack-free and highly aligned oxide buffer layers on textured metal substrates to protect the superconductors from metal contamination and, to provide an appropriate biaxially oriented, lattice matched substrate for the subsequent epitaxial growth of superconducting YBCO on top of it. Furthermore, such a barrier layer may also limit diffusion as well from the metal substrate to the superconducting coating as inversely, thereby allowing a better control of purity and oxygen content. Before a buffer layer can be deposited, the substrates must be prepared for coating by an optimized cleaning procedure. Subsequently, optimized buffer layer architectures must be designed in order to have a biaxially textured, smooth and dense thin film that fulfills all the requirements. Many buffer layers including CeO_2_, doped CeO_2_, YSZ, La_2_Zr_2_O_7_, Gd_2_Zr_2_O_7_, La_x_Ce_1−x_O_δ_ have been prevalently used for the support of YBCO growth [2,3,4,5,6,7,8,9,10,11,12,13]. Our work was focused on depositing water based buffer layers using chemical solution deposition method (CSD) on the Ni-5% W substrates. Water-based methods were considered because of their effectiveness based on environmental and economic reasons [7,8,9,10,11,12,13,14,15]. Since water-based solutions possess a quite high surface tension, an optimal way of preparing the substrate to get a uniform coating on top of it was a challenge in its own way. Following this, different cleaning methods with inclusion of chemicals had to be considered in order to improve wetting behavior. However, inclusion of chemicals can add-up to the already present impurities on the substrates in the form of carbon from the atmosphere. Moreover, grease and oil is used while rolling the substrates during its preparation for obtaining epitaxial RABiTS Ni-5% W substrates. This can also add to the level of carbon contaminations. Additionally, the substrates can be covered by a passivating layer of NiO on top, which can prove detrimental for the to-be deposited buffer layers in terms of transferring the epitaxy. Therefore, a suitable and convincing cleaning method had to be chosen and more fundamental insights on the surface behavior had to be gained.

Previously, there have been a few reports on different cleaning methods used for the preparation of Ni-5% W substrates for coating the buffer layers using CSD [6,10,11,16]. Sathyamurthy *et al.* [16] examined the effect of a chemical etching process on the amount of carbon and oxygen contamination on the nickel surface. 

In this work, the focus has been directed towards elucidation of the action of different cleaning methods on the Ni-5 at % W substrates, including a thermal treatment to make it suitable for coating of aqueous based buffer layers. The results obtained from a combination of a chemical and thermal cleaning procedure based on a sequence of chemical treatments in trichloroethylene, acetone, methanol, deionized water and an etching mixture of hydrogen peroxide and formic acid and thermal treatment at 800 °C are reported. The chemical composition in terms of atom percentages of Ni and W and the contaminants including C and O quantified using XPS is presented. Previously, XPS has been used as an effective analytical tool for studying the buffer layer capacity [17]. The wetting property of the substrate after different cleaning methods has been pictured and presented. It was found that the cleaning treatment significantly improves the wettability of the substrates, a crucial property for the epitaxial growth of crack-free and homogeneous buffer layers by means of water-based solution-gel deposition techniques.

## 2. Experimental Section

Flexible 80 µm thick and polished substrates were provided by D-Nano GmbH and produced by evico GmbH. They have been considered for investigation throughout this work.

### 2.1. Chemical Cleaning with Etching Process

The substrates were dipped consecutively in trichloroethylene 99% (Acros Organics), in acetone 99.5% (Fiers) and in methanol 99.85% (Fiers), each dipping step lasted for 5 min. This was carried-out in order to remove organic traces of oil, grease, and also dirt which can occur due to their use in rolling process of the RABiTS Ni-5% W substrate. This degreasing procedure was followed by a 5 min rinse in MiliQ water obtained from a MILLIPORE 00A/040 MiliQ water purification system. In this step, residual solvents and ionic salts were removed. Subsequently, the substrates were introduced in a hot mixture (50–55 °C) 50 vol % each of hydrogen peroxide (H_2_O_2_) 35% in weight (Sigma Aldrich) and formic acid (HCOOH) 99% (Chem-Lab), for 15 min. This final step will involve “etching” of the surface, more precisely the grain boundaries. Substrates were finally rinsed twice for 10 min with MiliQ water. This whole sequence of the treatment is called “chemical cleaning with etching” of substrates.

### 2.2. Thermal Cleaning Process

In the “thermal cleaning process” substrates were exposed to high temperatures (800 °C) for 1 h in a quartz tubular furnace (Carbolite, Three zone furnace) and in a reducing atmosphere provided by a continuous flow of Ar-5% H_2 _gas maintained at 0.2 L/min. The native NiO, which can act as passive protection for the Ni-5% W substrates will be reduced under the reducing gas flow during this step. Heating and cooling rate was set at 10 °C/min. After this thermal treatment the substrates were stored in methanol to avoid contamination from air and particulates in the atmosphere.

### 2.3. XPS Characterization

The contaminants, such as carbon and oxygen (as NiO), present in the tapes under uncleaned and differently cleaned conditions were evaluated by XPS depth profiling (S-probe, Surface Science Instruments, VG with a monochromatic AlK_α_-source, 1,486.6 eV). The voltage and power of the source were kept constant at 10 KV, 200 W. Sputtering of an area of 3 × 3 mm^2^ was performed with an Ar^+^-ion gun (4 keV). After each consecutive sputter cycle an area of 250 × 1000 µm^2^ was analyzed with an hemispherical analyzer. Regions for O 1s, C 1s, Ni 2p and W 4f peaks were registered with a resolution of 0.15 eV. Peak areas were converted into atomic concentrations with the software package CasaXPS (Casa Software Ltd., UK) using a Shirley background and Scoffield sensitivity factors. Ar^+^-ion sputtering was carried-out for four consecutive sputter cycles each lasting 10 s. In total, 40 s of sputtering was carried out. According to the standard sputtering rate of Ta_2_O_5_, fixed at 0.15 nm/s, each sputter cycle can be roughly estimated to remove 1.5 nm thickness of the substrate and in total, therefore approximately 6 nm thickness of the substrate is believed to have been sputtered and removed. 

The XPS study was supported by a reflection high energy electron diffraction (RHEED) analysis. RHEED is an electron diffraction technique used to characterize the topmost layer (2–5 nm) of a material. A focused beam of electrons, generated by an electron gun, strikes the surface of the sample under a grazing angle (<1.5°). The electrons are diffracted by the atoms at the sample’s surface. Due to the very small angle and a wavelength of 7.08 nm for an accelerating potential of 30 keV, their mean free path perpendicular to the surface is 5 to 10 nm at the most. Typically, the electrons penetrate small islands at a rough surface, which leads to a transmission pattern similar to transmission electron microscopy. The diffraction pattern, captured on a photo luminescent screen with an additional CCD camera, as a result exhibits either discrete spots (for a highly oriented material) or spots merged into rings (for polycrystalline material). The as-received and cleaned Ni-5% W substrates were analyzed using a STAIB RHEED instrument at the IFW Dresden, Germany. The top surface crystallinity with diffraction spots corresponding to epitaxial metallic Ni is crucial for the further deposition of buffer layers to grow *c*-axis oriented superconducting YBCO.

## 3. Results and Discussion

In the following discussion, different combinations of chemical and thermal cleaning were carried-out on the substrates and have been analyzed using XPS.

### 3.1. Uncleaned Ni-5% W Substrate

**Figure 1:**
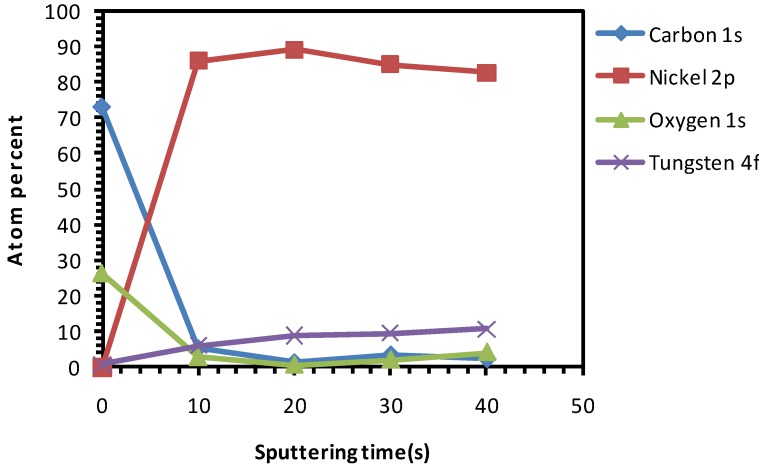
X-ray photoelectron spectroscopy (XPS) depth profile studies of uncleaned Ni-5% W substrate.

Figure 1 shows the depth profile XPS-study of the as-received, uncleaned substrate. The top surface shows a significant carbon contamination. The native carbon and oxygen contamination is removed after 10 s of sputtering, exposing the Ni and W peaks. 

**Figure 2:**
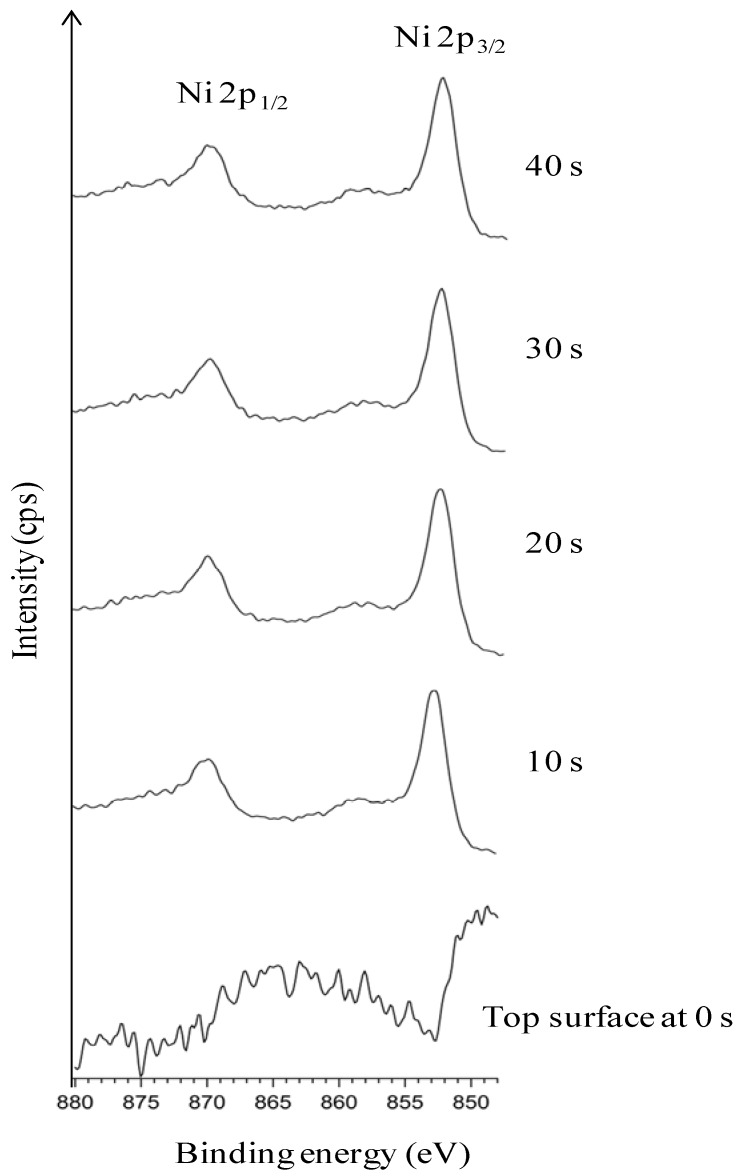
XPS depth profile studies of Ni peaks in uncleaned substrates.

Figure 2 explicitly depicts the Ni peaks under different sputtering cycles (from bottom to top). The Ni peaks in different sputtering cycle are shown. The top-surface exposing the epitaxial Ni is crucial for the epitaxial growth of the following buffer layers and YBCO growth. The intensity of Ni peaks on the top surface and in different depths of sputtering, correlates to the exposed epitaxial Ni surface. The intense Ni peaks using XPS (AlK_α_): Ni 2p_3/2_ and Ni 2p_1/2_ for metallic Ni is expected at a binding energy of 852.3 eV and 869.7 eV (along with their corresponding satellite peaks) whereas, it is expected at 853.3 eV and 871.7 eV for NiO. Normally, any observation of a broader and shifted peak at 853.3 eV can be correlated to the presence of NiO. Additionally, a peak shift in Ni peaks helps in verifying whether it corresponds to metallic Ni or that of NiO. 

**Figure 3:**
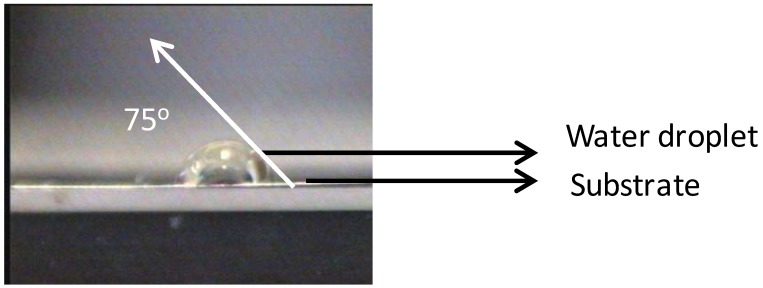
Non-wetting droplet of water on top of uncleaned Ni-5% W substrate.

As seen in Figure 2, almost no Ni is visible on the top-surface (0 s of sputtering). After sputtering Ni peaks can be correlated to the corresponding metallic Ni peak position as observed from the figure. The reason for the less intense peak of Ni can be attributed to the presence of surface carbon and oxygen as shown in Figure1. This shows the necessity of cleaning the surface prior to deposition of the buffer layers. The wettability of the substrate was evaluated by measuring the contact angle of water. It can be seen that, the water droplet did not wet the substrate, instead it remained stagnant with a contact angle of >75° (Figure 3). This also means that the wettability of the native substrate needs to be improved in order to coat it using water-based solutions [9,13].

### 3.2. Chemical Cleaning with Etching for 15 min

**Figure 4:**
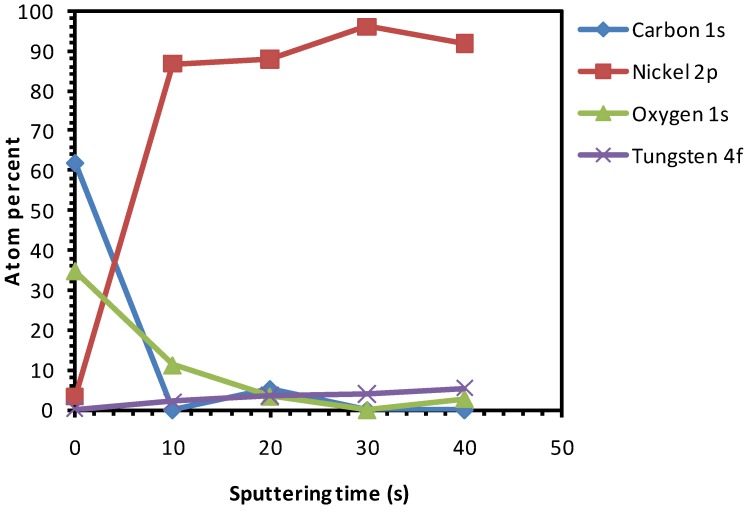
XPS depth profile studies of Ni-5% W substrate chemically cleaned with etching for 15 min.

The as-received substrates were chemically cleaned and etched for 15 min. Figure 4 depicts the various percentages of elements present in this sample after different sputter cycles, starting from the top-surface. On the surface of the sample a relatively high amount of oxygen was observed. This can be attributed to the use of the HCOOH-H_2_O_2_ mixture which releases active oxygen species that can attach to the surface of the sample. 

**Figure 5:**
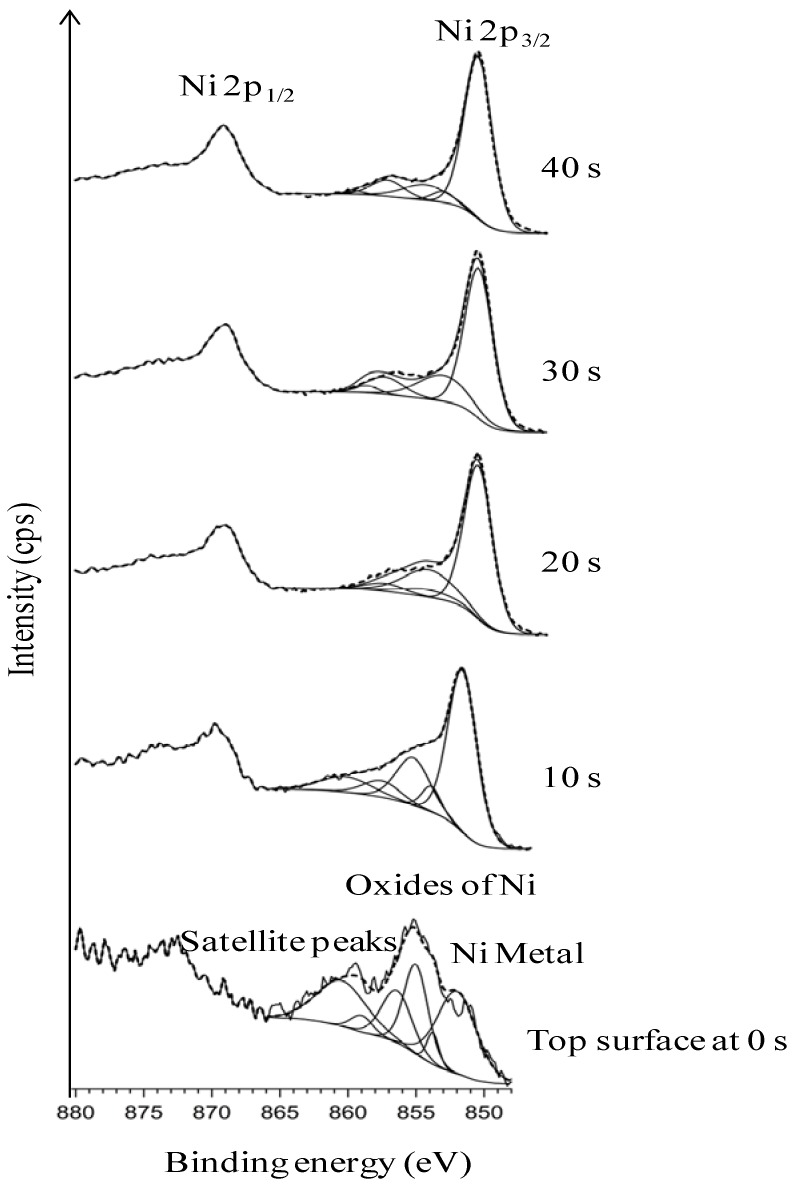
XPS depth profile studies of Ni peaks in substrate chemically cleaned with etching for 15 min (with deconvolution for the presence of NiO).

From Figure 4, the presence of oxygen can be seen even after 20 s of sputtering which indicates that the etchant has penetrated several nanometres deep into the substrate. From this we can conclude that the oxidation of the substrate up to ~4.5 nm thickness has occurred. The oxygen peaks, seen in Figure 4 after 10 s of sputtering, is indexed to be partially corresponding to that of NiO. Growth along the (111) phase is thermodynamically favorable for NiO. This is highly undesirable because it can induce growth of the buffer layer with a similar orientation, thus hampering the growth of c-axis oriented superconducting YBCO. Figure 5 shows the Ni-peak after different sputtering cycles.

Compared to uncleaned substrate, there is a higher amount of Ni that is exposed on the top-surface after this cleaning procedure. Also, a decrease in amount of C at the surface can be seen as compared to that of the uncleaned substrate (Figure 4 *vs.* Figure 1). The Ni 2p3/2 peak has been deconvoluted according to the guidelines of Biesinger *et al.* [18] with peaks for metallic and oxidized Ni, as well as for their satellites, From this graph we can see that the top surface is predominantly composed of oxidized Nickel with a peak at higher binding energies (above 853 eV), while with sputtering the peak at higher binding energies diminishes and the fraction of metallic Ni increases.

Additionally, it can be seen that, after etching, the concentration of carbon has decreased in comparison to uncleaned substrates (although the oxygen percentage is higher). This signifies that the carbon impurities are removed after etching. On the other hand, the oxygen percentage remained high, showing the formation of NiO as stated before, which is very much undesirable.

**Figure 6:**
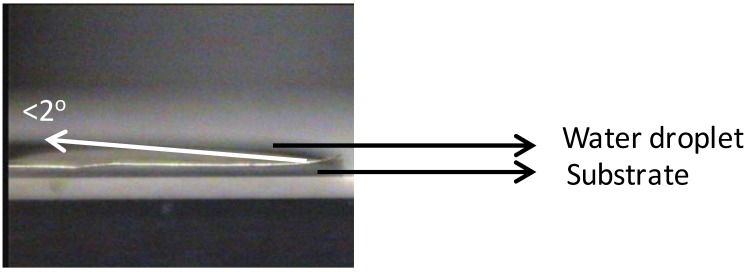
Perfectly wetted droplet of water on top of the substrate chemically cleaned with etching for 15 min.

However, chemical cleaning followed by etching for 15 min significantly improved the wettability of the substrate with a contact angle of <2° (Figure 6). This improvement in the wettability can be interpreted as an increased adhesion of water on the surface of the substrate which could have been caused by an increase in roughness of the substrate due to the formation of NiO (Figure 6).

### 3.3. Thermal Cleaning

**Figure 7:**
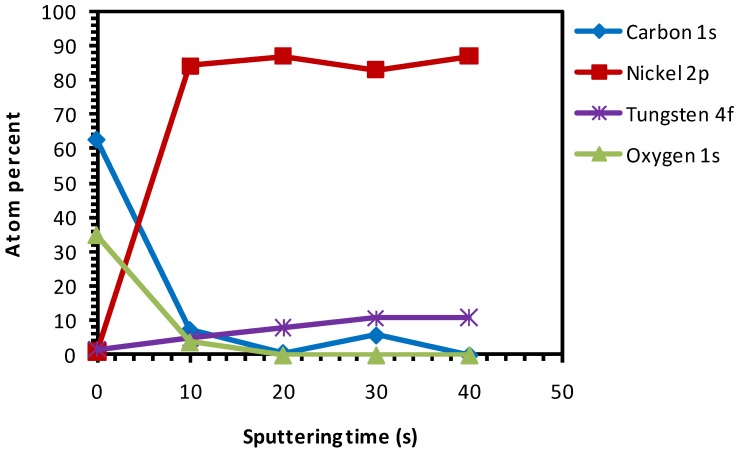
XPS depth profile studies of Ni-5% W substrate after thermal cleaning.

Thermal cleaning of the native substrates was carried-out under a continuous flow of reducing gas (Ar-5% H_2_) to remove any native NiO that could be present in the substrate. As seen in Figure 7, after thermal cleaning at 800 °C for one hour, surface oxygen is reduced but not totally removed. 

**Figure 8:**
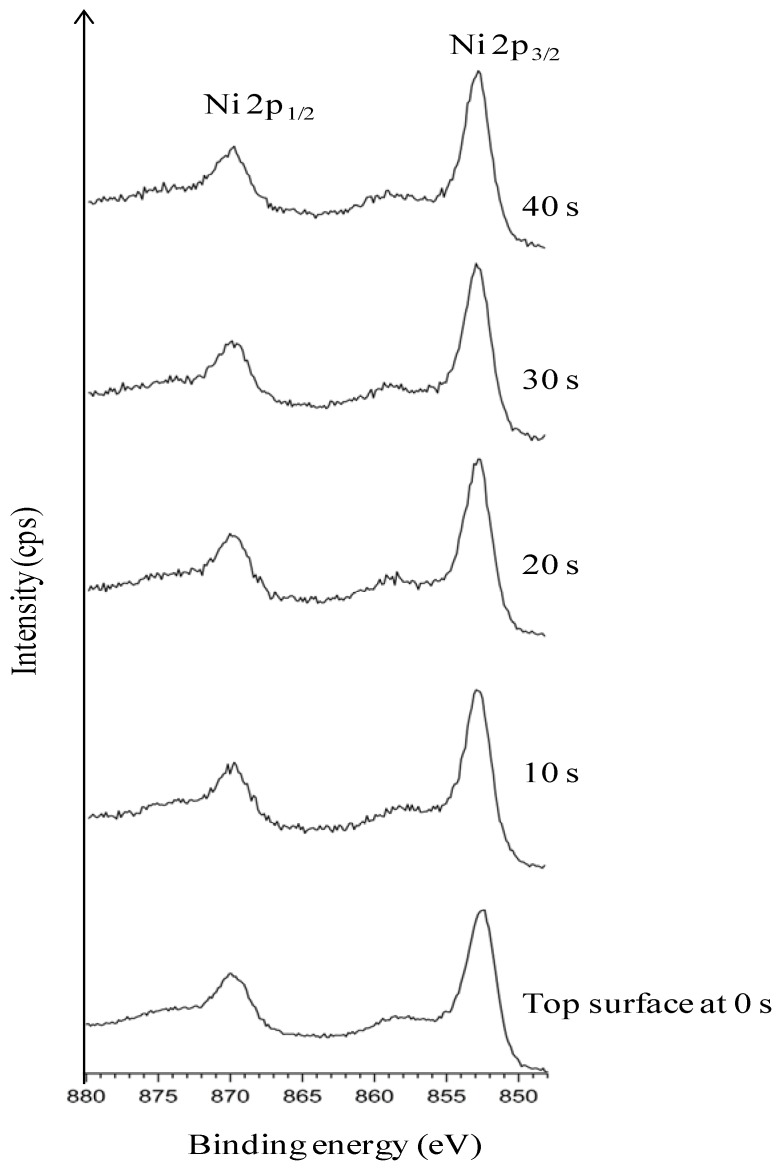
XPS depth profile studies of Ni peaks in substrate after thermal cleaning.

**Figure 9:**
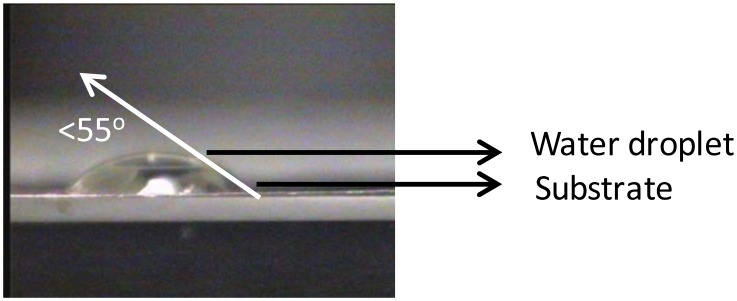
Water droplet on top of a thermally cleaned Ni-5% W substrate.

Figure 8 shows that the top-surface is revealing the Ni peak. The broad hump around 855 eV which was observed in Figure 5 and allocated to oxidized nickel is now absent. The wettability of the substrate was slightly improved as seen in Figure 9 (contact angle of <55°). 

Both the chemical and thermal cleaning methods resulted in a decrease of carbon content, as seen in the spectra of the top surface of the sample (Figure 4 and Figure 7, respectively). However, chemical etching resulted in a good wettability at the cost of introducing high amount of oxygen as observed in the inner part of the sample (Figure 4). On the other hand, the thermal process showed no increase in the oxygen content in the sample and only a minor improvement in the wettability (Figure 7 and Figure 9, respectively).

### 3.4. Thermal Cleaning Followed by Chemical Cleaning with Etching for 15 min

**Figure 10:**
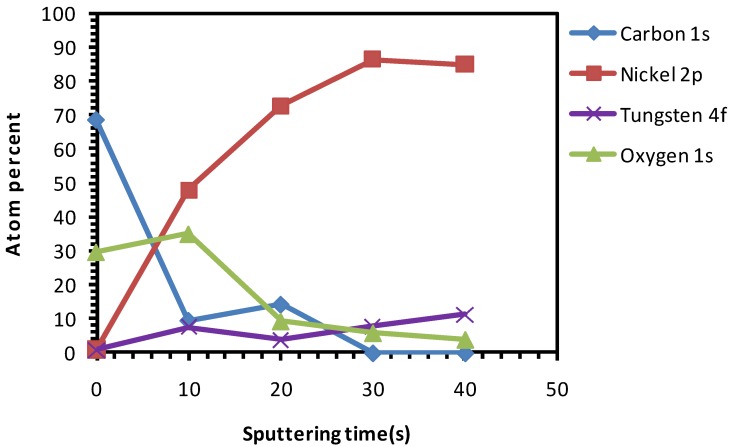
XPS depth profile studies of Ni-5% W substrate after thermal cleaning followed by chemical cleaning with etching for 15 min.

**Figure 11:**
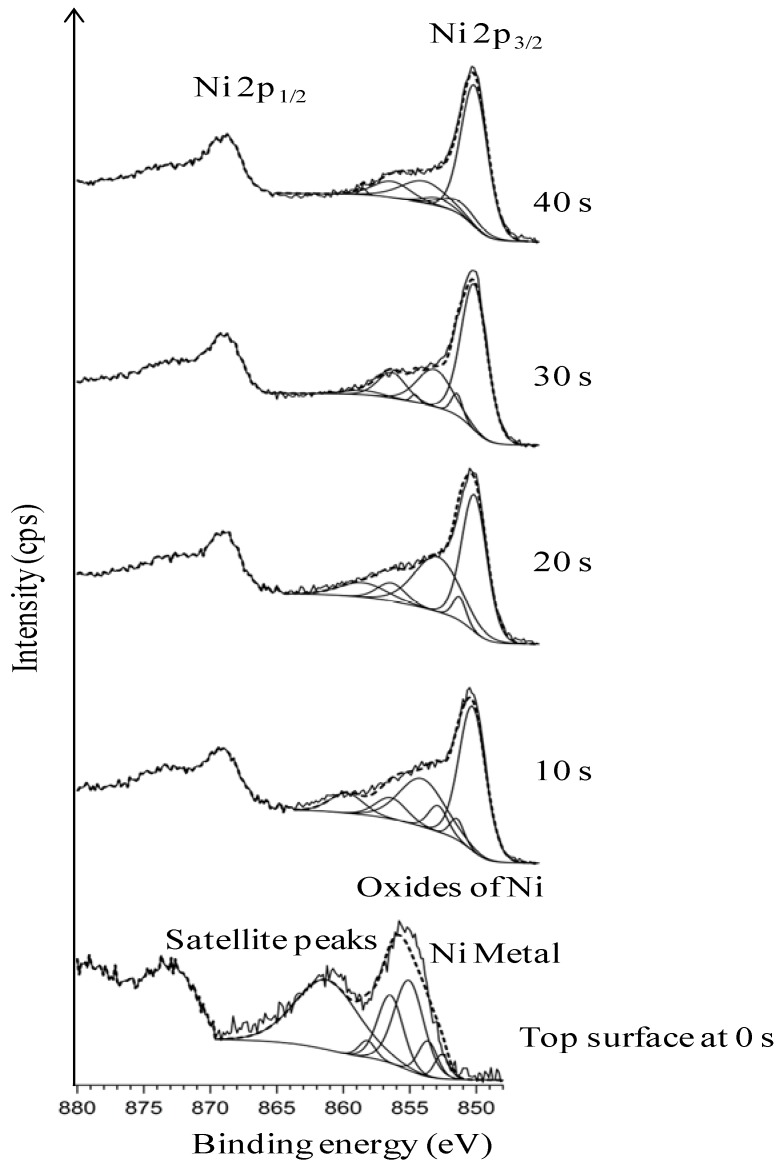
XPS depth profile studies of Ni peaks after thermal cleaning followed by chemical cleaning with etching for 15 min (with deconvolution for the presence of NiO).

To ensure good wettability of the surface the above said sequence of thermal treatment was followed by chemical cleaning and etching. This process resulted in an improvement in the wettability with a contact angle of <5°, but the contamination with NiO did not improve as seen in Figure 10 and Figure 11.

### 3.5. Chemical Cleaning with Etching for 15 min Followed by Thermal Cleaning

**Figure 12:**
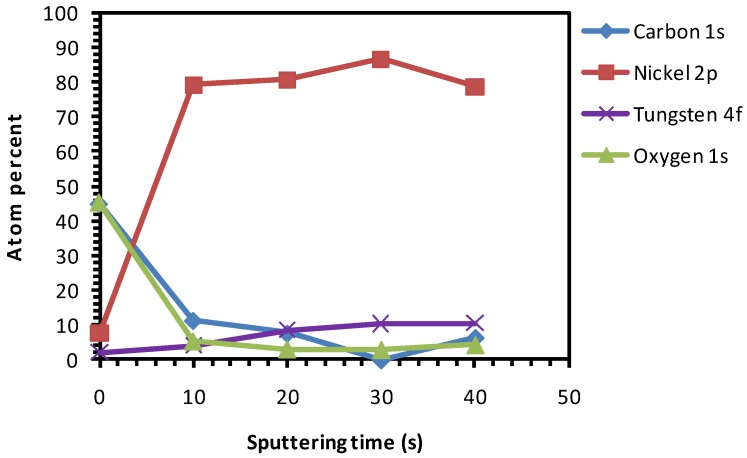
XPS depth profile studies of Ni-5% W substrate after chemical cleaning with etching for 15 min followed by thermal cleaning.

**Figure 13:**
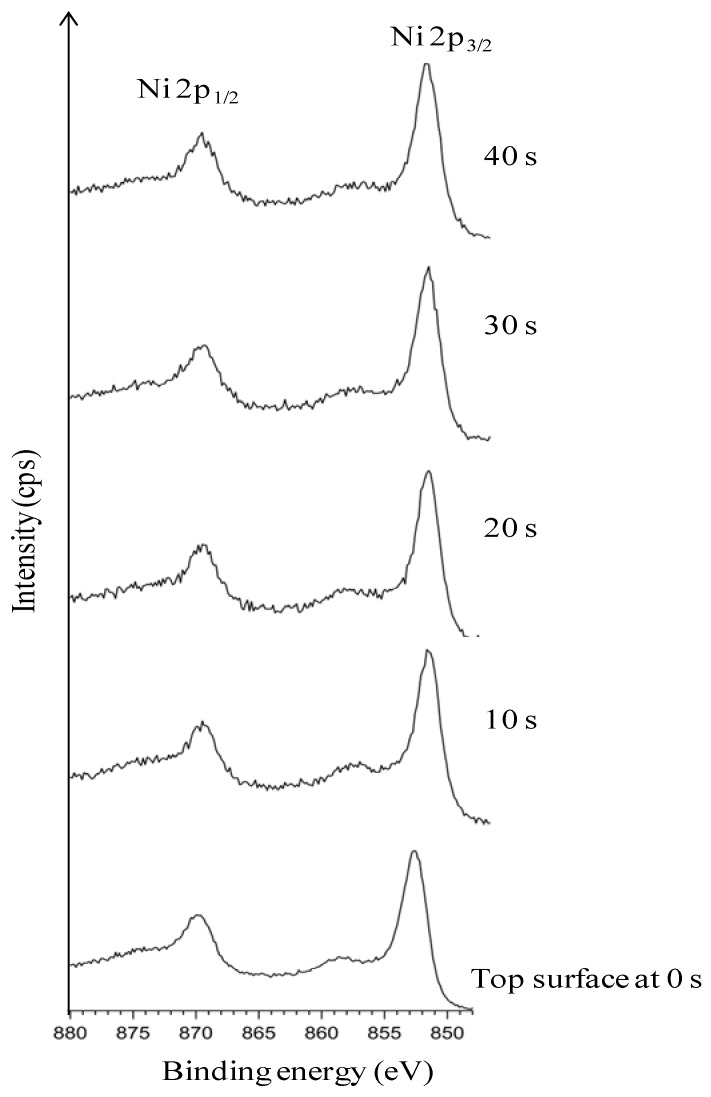
XPS depth profile studies of Ni peaks after chemical cleaning with etching for 15 min followed by thermal cleaning.

According to Sathyamurthy *et al.* [16], a chemical cleaning followed by ultrsonic cleaning in methanol improved the wettability and marginally reduced the carbon contaminations. According to Knoth *et al.* [6], cleaning the substrates in an acetone ultrasonic bath followed by thermal treatment under Ar-5% H_2_ showed good wettability. According to Cloet *et al.* [10], a thermal treatment of the substrates under reducing gas flow followed by chemical cleaning and etching improved the wettability. Since, the thermal treatment followed by chemical cleaning and etching showed significant oxidation, as seen in the previous section, the reverse procedure of chemical cleaning with etching followed by thermal cleaning was applied. As seen in Figure 12 and Figure 13, the Ni-peaks are visible on the top-surface and no oxidation of the substrate can be observed.

### 3.6. RHEED Analysis

**Figure 14:**
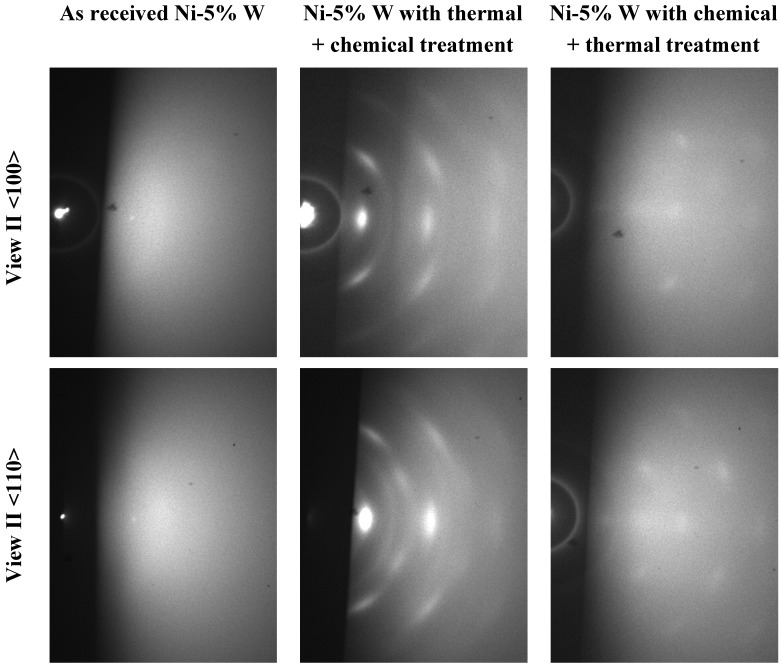
Reflection high energy electron diffraction (RHEED) pattern of uncleaned and differently cleaned Ni-5% W substrate.

Since top-surface crystallinity is crucial for epitaxial growth of the buffer layers and the YBCO layer thereon, the substrates were analyzed by RHEED after different cleaning procedures. Therefore, an uncleaned substrate, a substrate with thermal followed by chemically cleaning with etching and the substrate with chemical cleaning with etching followed by thermal cleaning was analyzed. The samples were aligned in such a way that the electron beam was parallel to the <100> or <110> direction of the cube textured Ni-5% W tape (Figure 14).

**Figure 15:**
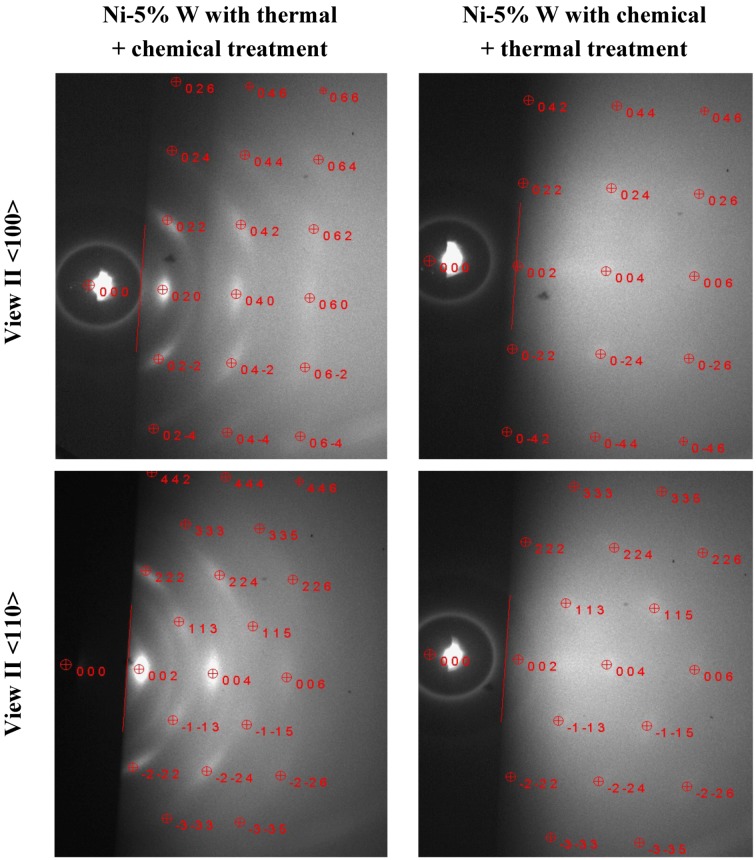
Comparison between the measured RHEED pattern and a simulation of the diffraction spots using NiO for sample with thermal followed by chemical treatment, whereas the pattern of the chemically followed by thermally treated substrate can be indexed with pure Ni.

The untreated RABiTS Ni-5% W tape shows only a diffuse diffraction pattern indicating an amorphous or nanocrystalline surface structure. Such patterns are typically observed on these substrates as a thin nanocrystalline untextured NiO is building up during storage even at room temperature. As a result, no crystalline RHEED spots from the Ni tape itself are observed. In contrast, both of the cleaned Ni-5% W tapes exhibit crystalline diffraction spots. The spots are clearly visible on the substrate after the thermal followed by chemical cleaning and somewhat more diffuse on the sample treated with an opposite cleaning sequence. The RHEED diffraction pattern was recalculated and indexed using software based on a kinematic diffraction theory in order to get information on the crystal structure of the surface layer. The results were compared to the measured pattern (Figure 15). 

The diffraction pattern of the sample, which was first cleaned thermally and afterwards chemically was identified to arise from a textured NiO layer at the surface (the spots were calculated with a lattice constant of 4.17 Å), which has a cube on cube epitaxial relationship. Such biaxial NiO textures were previously observed on different RABiTS tapes and are intentionally prepared in the surface oxidation epitaxy process [19,20]. Additionally, small polycrystalline rings are visible in the pattern, which might also origin from the NiO layer indicating that this layers shows different texture components. This identification of a top NiO layer confirms the results of the XPS measurements presented above (see Section 2.4). In contrast, the diffraction spots on sample treated with the opposite cleaning procedure fit well with the Ni lattice of the substrate materials itself having a lattice constant of 3.52 Å. This indicates that this cleaning treatment is most suitable for the epitaxial growth of a subsequent buffer layer.

In summary, the combination of chemical cleaning with etching for 15 min followed by thermal cleaning was chosen to be the best in terms of a reduced surface contamination, an undisturbed textured Ni surface and an improved wettability with a contact angle of 18° which is sufficient for dipcoating in an aqueous sol-gel system [17]. Such cleaned substrates are particularly suitable for water-based solutions, which have a high surface tension.

## 4. Conclusions

Different ways of cleaning the Ni-5% W substrate were tested. The contaminations of the surface in terms of carbon and oxygen were quantified using XPS and the best cleaning method was chosen based on a minimal contamination, and good surface wettability. Thus a chemical cleaning with etching for 15 min followed by thermal cleaning is preferred. This procedure results a in minimal contamination of the top-surface with carbon impurities and NiO, while providing a contact angle of <20°. An additional RHEED study on this substrate showed diffraction spots corresponding to cube textured Ni indicating a clean substrate surface. This is an important prerequisite for an epitaxial growth of buffer layers, in particular for the application of water-based precursor solutions.
